# Neurosurgical and Perioperative Management of Chronic Subdural Hematoma

**DOI:** 10.3389/fneur.2020.00550

**Published:** 2020-06-19

**Authors:** Jurre Blaauw, Bram Jacobs, Heleen M. den Hertog, Niels A. van der Gaag, Korné Jellema, Ruben Dammers, Hester F. Lingsma, Joukje van der Naalt, Kuan H. Kho, Rob J. M. Groen

**Affiliations:** ^1^Department of Neurology, University of Groningen, University Medical Center Groningen, Groningen, Netherlands; ^2^Center for Medical Decision Sciences, Department of Public Health, Erasmus Medical Center, Rotterdam, Netherlands; ^3^Department of Neurology, Isala Hospital Zwolle, Zwolle, Netherlands; ^4^University Neurosurgical Center Holland (UNCH), Leiden University Medical Center, Haaglanden Medical Center, Haga Teaching Hospital, Leiden, Netherlands; ^5^Department of Neurology, Haaglanden Medical Centre, Hague, Netherlands; ^6^Department of Neurosurgery, Erasmus MC Stroke Center, Erasmus Medical Center, Rotterdam, Netherlands; ^7^Department of Neurosurgery, Medisch Spectrum Twente, Enschede, Netherlands; ^8^Department of Neurosurgery, University of Groningen, University Medical Center Groningen, Groningen, Netherlands

**Keywords:** chronic subdural hematoma, neurosurgery, anesthesia, bilateral chronic subdural hematoma, logistic regression

## Abstract

**Objective:** Surgery and specifically burr hole craniostomy is the most common first choice treatment of patients with Chronic Subdural Hematoma (CSDH). However, several aspects of neurosurgical and peri-operative management are still a subject of research, such as how to treat bilateral CSDH and the anesthetic approach. We aim to investigate the effect of the surgical approach to bilateral CSDH and the effect of anesthesia modality on outcome of CSDH patients.

**Methods:** We retrospectively included surgically treated CSDH patients between 2005 and 2019 in three hospitals in the Netherlands. The effect of the surgical approach to bilateral CSDH (unilateral vs. bilateral decompression) and anesthesia modality (general vs. local anesthesia) on outcome (complications, recurrence, and length of hospital stay over 4 days) was studied with logistic regression adjusting for potentially confounding radiological and clinical characteristics.

**Results:** Data of 1,029 consecutive patients were analyzed, mean age was 73.5 years (±11) and 75% of patients were male. Bilateral CSDH is independently associated with an increased risk of recurrence within 3 months in logistic regression analysis (aOR 1.7, 95% CI: 1.1–2.5) but recurrence rate did not differ between primary bilateral or unilateral decompression of bilateral CSDH. (15 vs. 17%, *p* = 0.775). Logistic regression analysis showed that general anesthesia was independently associated with an increased risk of complications (aOR 1.8, 95% CI: 1.0–3.3) and with a length of hospital admission of over 4 days (aOR 8.4, 95% CI: 5.6–12.4).

**Conclusions:** Bilateral CSDH is independently associated with higher recurrence rates. As recurrence rates in bilateral CSDH are similar for different surgical approaches, the optimal choice for primary bilateral decompression of bilateral CSDH could vary per patient. General anesthesia for surgical treatment of CSDH is associated with higher complication rates and longer hospital admission.

## Introduction

Chronic subdural hematoma (CSDH) is one of the most frequent neurosurgical diseases with an incidence of about 17/100,000/year, increasing with age (1). CSDH is often regarded as a benign, easily treatable disorder, but recurrence rates vary between 10 and 15% and mortality can be as high as 27% (2). The most commonly reported risk factors for CSDH are: older age, use of anticoagulants, male sex, alcoholism, and a history of direct or indirect head trauma (3, 4). Although surgical treatment remains first choice, conservative treatment modalities are more and more implemented in daily clinical practice (5). The optimal surgical technique has been studied extensively (6–8), resulting in burr hole craniostomy (BHC) with (subdural) drainage being the preferable method (5, 9–11). Other aspects of neurosurgical and perioperative management of CSDH are less well studied and evidence is still lacking. In this study, we focus on two specific neurosurgical and perioperative aspects of CSDH treatment on which uncertainty exists; the surgical approach to bilateral CSDH and type of anesthesia modality.

There is no consensus about the optimal surgical approach of bilateral CSDH, even though about 25% of CSDH is bilateral at presentation (12, 13). Reports on recurrence rates of bilateral and unilateral decompressed bilateral CSDH vary, and some studies have shown a beneficial effect of primary bilateral decompression in bilateral CSDH (10, 13, 14). However, currently the choice of unilateral or bilateral decompression mainly depends on lateralizing of symptoms and hematoma characteristics (13, 15). Studies that have reported a benefit from primary bilateral decompression suggest that the recurrence is related to a decrease in intracranial pressure after unilateral decompression and subsequent shift of the brain toward the operated side (13). Both factors might lead to an increase of the contralateral subdural space and subsequent expansion of the contralateral hematoma, rather than actual recurrence.

To address the uncertainties concerning the surgical approach of bilateral CSDH, we compare recurrence rates in bilateral and unilateral CSDH, assess the possible beneficial effect of primary bilateral decompression and study the factors associated with choice for side of decompression. We hypothesize that there is a beneficial effect of primary bilateral decompression in reducing the recurrence rate of bilateral CSDH.

The choice of the optimal anesthesia modality is an important aspect of perioperative and neurosurgical management of CSDH. In other conditions primarily affecting elderly patients, the benefits of localized anesthesia (LA) over general anesthesia (GA) has been reported (16). For CSDH only limited studies on this subject exist. Studies that have assessed the differences between LA and GA in CSDH, report beneficial effects of LA (17, 18). Despite this finding, a recent large prospective study showed that 93% of CSDH patients is treated with GA (10).

We hypothesize that there is a beneficial effect of primary bilateral decompression compared to unilateral decompression on recurrence of bilateral CSDH and that LA compared to GA reduces postoperative complication rates and lowers length of hospital stay for patients with CSDH.

## Methods

We retrospectively collected data of all consecutive CSDH patients that were treated in three Neurosurgical centers in the Netherlands between 2005 and 2019. The inclusion period per center varied, based on the availability of patient data caused by transitions from paper to electronical patient files, from eight to thirteen years. We identified patients based on the codes registered for diagnosis, treatment and operation. Exclusion criteria were: Age under 18 years and acute subdural hematomas, defined as a SDH consisting of more than 1/3rd hyperdense components. Furthermore, patients with ventriculo-peritoneal shunts, patients with a history of intracerebral tumor or arteriovenous malformation prior to CSDH diagnosis were excluded as well as all patients who did not receive surgical treatment for their CSDH. The study was approved by the local ethical committees of the three neurosurgical centers. As all the data had been collected for routine clinical care, the necessity of informed consent was waived for this retrospective study.

We recorded side hematoma and of operation, together with anesthesia modality. Specifically we compared unilateral vs. bilateral decompression in patients with bilateral CSDH and general vs. local anesthesia.

Clinical and demographic data that were recorded included age, sex, comorbidity, medication use, hospital of treatment, and length of hospital stay. Additionally, we collected data on clinical severity measured by Markwalder Grading Scale (19) (MGS) and Glasgow Coma Scale (GCS)and on admission. Comorbidities at time of diagnosis were measured with the Charlson Comorbidity Index (CCI) (20).

Preoperative data included CT data comprising hematoma characteristics, such as type [classification of Nakaguchi et al. (21)] side of hematoma, maximal hematoma thickness in the axial plane (in centimetres) and midline shift (in millimetres).

Outcomes were complications, recurrence and length of hospital stay. Recurrence was defined as a return of clinical symptoms and reaccumulation of the CSDH on imaging (CT-scan), requiring retreatment (of the hematoma) within three months from the initial diagnosis. Postoperative complications included delirious state, rebleeding, wound infection, seizures, and systemic infection and were scored to be either present or absent. Due to low numbers of individual postoperative complications, complications were grouped and dichotomized into present or absent. Length of hospital stay was dichotomized at the median of 4 days.

### Statistical Analysis

Baseline characteristics and outcomes were compared between intervention groups (unilateral vs. bilateral and local vs. general anesthesia) either with χ^2^ tests or Mann-Whitney *U*-test, depending on the type of the variable.

In multivariable binary logistic regression we tested the association of intervention group with outcome, adjusted for the potential confounders (age, sex, CCI, and MGS at presentation). Additionally, since length of stay and complications are correlated, we adjusted for length of hospital stay in the models for complications and for complications in the model for length of hospital stay. Finally for local vs. general anesthesia, we also adjusted for location of treatment (which hospital).

## Results

### Patient characteristics

We included a total of 1,029 patients, of whom 773 were men (75%). The mean age was 74 years (±11) (Table 1). A history of head trauma was present in 571 patients (55%). Most patients had a MGS of 1 or 2 (respectively, 32 and 64%) with a median GCS score of 15 (IQR: 1).

**Table 1:** General characteristics of 1,029 surgically treated CSDH patients.

**Variables**	**Patients, *N* (%)**	
Sex		
- Male	773 (75)	
- Female	256 (25)	
Age (mean ± SD)	73.5 (± 11)	
Head trauma prior to CSDH	571 (55)	
No/does not recall	256 (45)	
MGS score at presentation:		
MGS 0:	4 (0.4)	
MGS 1:	326 (32)	
MGS 2:	648 (64)	
MGS 3:	31 (3)	
MGS 4:	3 (0.3)	
Unknown	17 (1.7)	
GCS at presentation, median (IQR)	15 (1)	
Side of hematoma		
- Unilateral	772 (75)	
- Bilateral	257 (25)	
Use of Antithrombotic drugs:		
- None	436 (43)	
- Antiplatelet therapy	247 (24)	
- Anticoagulants	324 (32)	
- Other	18 (1)	
- Unknown	4 (0.4)	
Type of antiplatelet therapy		
- Acetylsalicylic acid	176 (71)	
- Clopidogrel	19 (8)	
- Dual therapy	52 (21)	
Type of anticoagulants		
- 310 (96)		
- DOAC	14 (4)	
Hematoma type^*^	Left (*N* = 681)	Right (*N* = 605)
- Hyperdense	34 (3)	36 (4)
- Isodense	131 (13)	129 (13)
- Separated	58 (6)	54 (5)
- Gradation	43 (4)	41 (4)
- Laminar	40 (4)	32 (3)
- Trabecular	145 (14)	121 (12)
- Hypodense	105 (10)	82 (8)
- Unknown	125 (12)	110 (11)
Hematoma thickness, cm (mean ± SD)	Left: 1.8 (± 0.7)	Right: 1.8 (± 0.7)
Midline shift, mm (mean ± SD)	8.2 (± 4.9)	
Type of surgery		
BHC	995 (97)	
Craniostomy	16 (1.5)	
TDC	6 (0.5)	
Unknown	11 (1)	
Anesthesia modality		
- Local	609 (66)	
- General	314 (34)	
- Unknown	106 (10)	
Length of hospital stay, days median (mean, range)	4 (8, 0–76)	
Postoperative complications	117 in 111 patients (11)	
Type of postoperative complication		
- Delirious state	34 (29)	
- Pneumencephalus	22 (18)	
- Empyema/wound infection	15 (13)	
- Seizures	14 (12)	
- Bleeding of operation wound	11 (9)	
- Systemic infection	8 (7)	
- Thrombosis /embolism	4 (3)	
- Other (e.g., aphasia, CSF leakage, traumatic subarachnoidal hemorrhage resulting from surgery)	9 (8)	
Recurrence <3 months	115 (11)	
Mortality <3 months	40 (4)	

* *Including bilateral hematomas*.

Regarding CSDH characteristics, 772 (75%) of patients had a unilateral hematoma and bilateral CSDH was present in 257 patients (25%). Antiplatelet therapy was used by 247 patients (24%) and 324 patients (32%) used oral anticoagulants. Acetylsalicylic acid (ASA) and Vitamin K Antagonists (VKA) were most often used, in 176 (71%) and 310 (96%), respectively.

Most common hematoma types were hypodense, isodense, and trabecular. As for surgical technique, BHC was applied in 995 patients (96%). Localized anesthesia was performed in 609 patients (66%). The median length of hospital stay was 4 days (IQR 8). A total of 117 postoperative complications occurred in 111 patients (11%). The most often seen complications were delirious state, pneumencephalus, empyema, or wound infection and seizures. Recurrence within 3 months of diagnosis occurred in 115 patients (11 %) and 40 patients died within three months (4%).

### Neurosurgical Centers

The data of three neurosurgical centers was used for this study. The majority of patients, 641 (62%) was treated in Center 1 (Table 2). The surgical technique used for drainage of CSH was similar in all three centers, i.e., burr-hole drainage in respectively, 99, 94, and 93% of patients with per-operative flushing/ irrigation of the hematoma compartment with saline. Median time of post-surgery drainage was 24 h in center 1 and 48 h in center 2 and 3. In center 2, a relatively high number of patients, 22 (15%) did not receive post-surgery drainage. In center 1, local anesthesia was applied in 587 (92%) of patients, whereas center 2 and 3 applied local anesthesia in, respectively, 2 (3%) and 20 (6%) of their patients.

**Table 2:** Overview of neurosurgical and perioperative management in the three neurosurgical centers.

	**Center 1** ***N* (%)** **Total = 641**	**Center 2** ***N* (%)** **Total = 69**	**Center 3** ***N* (%)** **Total = 319**
Treated with Burr hole craniostomy *N* (%)	634 (99)	65 (94)	296 (93)
Hours of post-surgery drainage (median)	24	48	48
No post-surgery drainage *N* (%)	14 (2)	15 (22%)	7 (2)
Local Anesthesia applied *N* (%)	587 (92)	2 (3)	20 (6)

### Bilateral CSDH

The recurrence rate in bilateral CSDH (40 patients, 16%) was significantly higher than in unilateral CSDH (75 patients, 10%) patients (*p* = 0.01). No differences were present in sex (*p* = 0.279), age (*p* = 0.555), MGS at admission (*p* = 0.279), CCI at diagnosis (*p* = 0.524) or three-month mortality rates (*p* = 0.139) (Table 3). In multivariable analysis, patients with bilateral CSDH were more likely to develop recurrence than those with unilateral CSDH, after adjusting for age, sex, MGS, and CCI at admission. (aOR 1.7, 95% CI: 1.1–2.5)

**Table 3:** Statistics of differences between unilateral and bilateral CSDH (*N* = 1029).

	**Unilateral CSDH** **N (%)** **Total = 772**	**Bilateral CSDH** **N(%)** **Total = 257**	***p*-value**
Sex			*0.279^1^*
- Male	573 (74)	200 (78)	
- Female	199 (26)	57 (22)	
Age (mean + SD)	73 (11.2)	74 (10.3)	*0.555^2^*
Markwalder Grading Scale at admission			*0.279^1^*
MGS 0:	4 (0.5)	0 (0)	
MGS 1:	247 (32)	79 (31)	
MGS 2:	486 (63)	162 (63)	
MGS 3:	21 (3)	10 (4)	
MGS 4:	1 (0.1)	2 (1)	
Missing	13 (2)	4 (1)	
Charlson comorbidity index			*0.524^1^*
CCI : 0	20 (3)	4 (2)	
CCI: 1	42 (5)	10 (4)	
CCI: 2	95 (12)	28 (11)	
CCI: 3	177 (23)	49 (19)	
CCI: 4	191 (25)	69 (27)	
CCI: 5	116 (15)	47 (18)	
CCI: 6 or more	128 (17)	45 (18)	
Missing	3 (0.4)	5 (2)	
Recurrence <3 months	75 (10)	40 (16)	*0.012^1^*
Mortality <3 months	26 (3)	14 (5)	*0.139^1^*

1 analyzed with χ^2^ test;

2 *analyzed with Mann Whitney U-test*.

**Figure 1:**
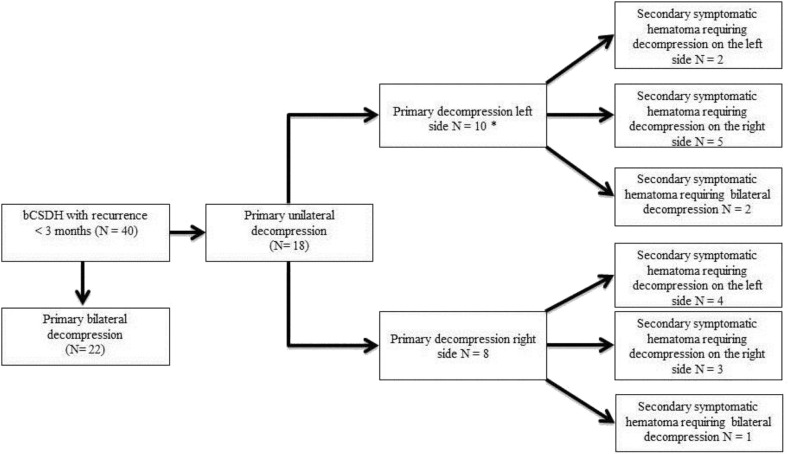
Numbers of contralateral decompression in bilateral CSDH with recurrence. *side of secondary hematoma requiring decompression is unknown in one patient.

We found no differences in recurrence rates between bilateral CSDH patients who underwent primary unilateral decompression (18 patients, 17%) and those who underwent bilateral decompression (22 patients, 15%, *p* = 0.77) (Table 4). Also, there was no statistically significant difference in hematoma thickness between patients with bilateral (2.9 cm. ± 0.8) and unilateral CSDH (3.0 ± 0.9, *p* = 0.057). Preoperative midline shift was found to be significantly larger in unilaterally treated bilateral CSDH (7.5 vs. 5.6 mm, *p* = 0.001). MGS at presentation did not differ (*p* = 0.66). We did find that in 50% (9 of 18) of the primary unilateral decompressed bCSDH with recurrence, the contralateral side was operated (Figure 1).

**Table 4:** Statistics of differences between unilaterally or bilaterally primary decompression in patients with bilateral CSDH.

	**Unilateral** **Decompression** ***N* (%)** **Total = 109**	**Bilateral** **Decompression** ***N* (%)** **Total = 145**	***p*-value**
Recurrence <3 months	18 (17)	22 (15)	*0.771^1^*
Hematoma thickness, cm (mean ± SD)	2.9 ± 0.8	3.0 ± 0.9	*0.057^2^*
MGS score at presentation:			*0.659^1^*
MGS 0:	0 (0)	0 (0)	
MGS 1:	30 (28)	47 (32)	
MGS 2:	73 (67)	88 (61)	
MGS 3:	3 (3)	7 (5)	
MGS 4:	1 (1)	1 (1)	
Missing	2 (2)	2 (1)	
Midline shift, mm (mean ± SD)	7.5 ± 3.8	5.6 ± 3.0	*0.001^2^*

*Number of bilateral CSDH patients with known side of primary decompression n = 254*.

1 analyzed with χ^2^ test;

2 *analyzed with Mann Whitney U test*.

### Local vs. Generalized Anesthesia

Anesthesia modality was known in 923 patients. The number of postoperative complications was significantly higher in general anesthesia (52 cases, 17%) than in local (50 cases, 8%) (*p* = 0.000) (Table 5) Median length of hospital stay was significantly longer in general anesthesia (8 days, IQR 8) than in local (3 days, IQR:3) (*p* = 0.000)and MGS and CCI at admission significantly differed between the groups (respective *p*-values 0.000 and 0.035). Age (*p* = 0.468) and sex (*p* = 0.085) were not significantly different. Also, no significant differences in three-month recurrence rates (12 vs. 10%, *p* = 0.510) and three-month mortality (4 vs. 3%, *p* = 0.324) between local and general anesthesia were present.

**Table 5:** Statistics of differences between local and general anesthesia.

	**Local anesthesia** ***N* (%)** **Total = 609**	**General anesthesia** ***N* (%)** **Total = 314**	***p*-value**
Sex (male)	464 (76)	232 (74)	*0.468^1^*
Age (mean + SD)	74 (10.7)	73 (11.4)	*0.085^2^*
MGS at admission			*0.000^1^*
MGS 0:	2 (0)	2 (0)	
MGS 1:	121 (20)	147 (47)	
MGS 2:	470 (77)	140 (45)	
MGS 3:	15 (3)	15 (5)	
MGS 4:	0 (0)	3 (1)	
Missing	1 (0)	7 (2)	
Charlson comorbidity index			*0.035^1^*
CCI : 0	10 (2)	11 (4)	
CCI: 1	32 (5)	13 (4)	
CCI: 2	68 (11)	42 (13)	
CCI: 3	152 (25)	57 (18)	
CCI: 4	161 (26)	72 (23)	
CCI: 5	96 (16)	53 (17)	
CCI: 6 or more	88 (15)	62 (20)	
Missing	2 (0)	4 (1)	
Length of hospital stay in days median (mean, range)	3 (5, 0-40)	8 (8, 1-76)	*0.000^2^*
Postoperative complication *N* (%)	50 (8)	52 (17)	*0.000^1^*
Recurrence <3 months *N* (%)	72 (12)	32 (10)	*0.510^1^*
Mortality <3 months *N* (%)	26 (4)	10 (3)	*0.477^1^*

*Number of patients with known anesthesia modality N = 923*.

1 analyzed χ^2^ test;

2 *analyzed with Mann Whitney U test*.

In multivariable analysis, patients who received general anesthesia were 1.8 times more likely to develop postoperative complications than those receiving local anesthesia. (aOR 1.8, 95% CI: 1.0–3.3) (Table 6). After adjusting for hospital of treatment, this relationship was no longer present. (aOR 1.7, 95% CI: 0.7–4.0) (Table 7).

**Table 6:** Relation between general anesthesia and postoperative complication and chance of hospital admission of five days or more.

	**Postoperative complication aOR (95% CI)^1^**	**Length of hospital stay five days or more aOR (95% CI)^2^**
General anesthesia	1.8 (1.0–3.3)	8.1 (5.4–12.1)

1 *Adjusted for age, sex, MGS at admission, Charlson comorbidity index, and length of hospital stay*.

2 *Adjusted for age, sex, MGS at admission, Charlson comorbidity index and occurrence of postoperative complication*.

**Table 7:** Relation between general anesthesia and postoperative complication and chance of hospital admission of five days or more.

	**Postoperative complication aOR (95% CI)^1^**	**Length of hospital stay five days or more aOR (95% CI)^2^**
General anesthesia	1.7 (0.7–4.0)	3.0 (1.6–5.6)

1 *Adjusted for age, sex, MGS at admission, Charlson comorbidity index, length of hospital stay and treatment hospital*.

2 *Adjusted for age, sex, MGS at admission, Charlson comorbidity index, occurrence of postoperative complication and treatment hospital*.

401 patients (51%) had a length of stay of 4 days or less, and 390 patients (49%) stayed 5 days or more. Patients who received general anesthesia were 8.1 times more likely to have a length of stay of more than four days. (95% CI: 5.6–12.1) (Table 6). When adjusted for hospital of treatment, this association was lower, but still present. (aOR 3.0 95% CI: 1.6–5.6) (Table 7).

## Discussion

In this large retrospective study, one of the largest to date, an independently higher chance of recurrence was found in patients with bilateral CSDH compared to patients with unilateral CSDH. However, a beneficial effect of primary bilateral decompression on reducing recurrence rates in bilateral CSDH, as we hypothesized was not found. Even though no significant differences were present, the number of unilateral treated patients that required secondary contralateral surgery for their recurrence, was high: about half of all patients. This increased chance of recurrence combined with the high number of contralateral treatments for recurrence do suggest that bilateral CSDH requires other treatment strategies, as is proposed by other authors (13).

The contrast of our results with the findings of a recent study stating that there is a beneficial effect of primary bilateral decompression, can be explained by the overall higher incidence of recurrence in their study (13). The authors do not provide an explanation for their high incidence of recurrence, but it could be related to the large number of patients not receiving subdural drainage, which is a risk factor for recurrence (6). In their study, patients that received conservative treatment were also included, opposed to our patients who all underwent surgery. Interestingly, they report a high number of primary craniotomies, which has been related to lower recurrence rates (22). In the literature the consensus is to reserve craniotomy for solid hematomas with multiple recurrences (23). It is debatable whether contralateral recurrence in bilateral CSDH should be considered as secondary hematoma growth rather than recurrence, however we classified it as recurrence in our study. This allowed us to compare our results with other studies, which also consider contralateral growth in bilateral CSDH as “recurrence” (13, 14).

Another important question was the type of anaesthesia that was used. Our study showed that general anesthesia is independently associated with higher odds of postoperative complications and prolonged hospitalization in patients with CSDH. However, this association decreased when adjusted for the hospital in which the surgery was performed. This is probably related to the fact that the vast majority of patients with local anesthesia (92%) was operated in only one of the three included neurosurgical facilities. There was no difference in (co)morbidity or clinical severity of patients between the hospitals, meaning that the reported differences in hospital stay and postoperative complications are not due to a selection bias. The skewed distribution was caused by the standard procedures of the neurosurgical facilities. For example in center 1, where the vast majority of LA is applied, CSDH patients are always operated under LA unless they are not cooperative or otherwise not having a healthy condition for LA. Contradictory, the other two neurosurgical facilities apply GA as part of their standard procedure and only perform surgery under LA if patient characteristics require so. However, in center 2, a relatively high percentage of patients was treated without application of a subdural drain for post-surgery drainage. This might explain the decreased relationships of anesthesia type with postoperative complications and length of hospital stay, after adjusting for hospital of treatment, where the use of subdural drainage is reported to diminish recurrence and therefore possibly reduces complications (24).

In the current literature, limited numbers of studies have been performed to investigate the difference between LA and GA in CSDH. A recent review article concluded that ‘For now, the anesthetist and surgeon, in consultation with the patient, must decide which anesthetic technique to use on an individual basis’ (25). However, some remarks can been made regarding the studies that have been cited by these authors. In a retrospective study including 1,000 CSDH patients, of which 919 received LA, no difference in outcome between the types of anesthesia was found (23). However, in this study outcome was only defined as “good vs. bad postoperative results” based on the MGS. Moreover, these LA patients underwent sedation with LA, and not LA alone. Another smaller and retrospective study reported more cardiac complications in the GA group, leading to a longer hospital stay (17). This article is only available in Korean, so the specific methods and possible limitations could not be retrieved. Our number of postoperative complications (without recurrence and death) may be perceived as being high, but is comparable to reported numbers in other CSDH studies (11, 26).

Not all studies share this view on the benefits of LA over GA. Some attribute the increased numbers of postoperative cognitive changes not to GA but to other aspects of surgery, such as an increase in cytokines, and question the supposed benefits of LA (27, 28). Other possible complicating factors of LA, described in the literature comprise discomfort for the patient and movement of patients during surgery, thereby increasing the chance of surgical complications (28, 29). When evaluating the majority of studies including our own results, we would like to conclude that LA, with the possibility to convert to GA in cases where patients are not cooperative, seems to be the better option for treatment of CSDH.

Despite the interesting results, this study comprises strengths and limitations. The strengths of our current study are a high number of patients, with data collected in multiple neurosurgical centers. The retrospective nature of our study may lead to a risk of selection bias, as only those patients who are deemed suitable or even fit for operation are referred to neurosurgical centers. This is partially illustrated by the low number of patients in the MGS 0, 3, and 4 groups. As a result we can mainly analyze the “milder” MGS 1 or 2 groups, possibly leading to lower complication and recurrence rates. The low complication numbers in our study can also be due to the fact that patients are transferred back to their own region quickly after surgery and consequently not all complications are registered in the patient charts of the neurosurgical departments. Finally, due to the observational nature of our study, randomized data was not available, possibly leading to confounding by indication. This is especially difficult seeing that we compared interventions. As a result observed and unobserved patient characteristics, e.g., CT findings, GCS, may drive real-life clinical decisions, confounding the relation between the intervention and the outcome. Statistical approaches can adjust for observed confounders but not for unobserved confounders so findings need to be interpreted with caution.

## Conclusion

Bilateral CSDH is independently associated with higher recurrence rates, but there is no difference in recurrence between primary bilateral decompression and unilateral decompression.

Our study suggests a beneficial effect of local anesthesia compared to general anesthesia. Therefore, in suitable patients, local anesthesia may be considered over general. Our findings regarding the optimal surgical approach in bilateral CSDH and the benefits of local anesthesia in CSDH should be confirmed in prospective studies.

## Data Availability Statement

Requests for access to the anonymized dataset can be send to the corresponding author.

## Ethics Statement

The studies involving human participants were reviewed and approved by Medical Ethical Review Board of the University Medical Center Groningen. Written informed consent for participation was not required for this study in accordance with the national legislation and the institutional requirements.

## Author Contributions

JB: conception and design, acquisition of data, analysis and interpretation of data and drafting the article, approved the final version of the manuscript on behalf of all authors. BJ, JN, and RG: conception and design, critically revising the article, reviewed submitted version of manuscript, study supervision. HH, NG, KJ, RD, HL, and KK: critically revising the article, reviewed submitted version of manuscript.

## Conflict of Interest

The authors declare that the research was conducted in the absence of any commercial or financial relationships that could be construed as a potential conflict of interest.

## Abbreviations

ASA: Acetylsalicylic acid
BHC: Burr-hole craniostomy
CCI: Charlson comorbidity index
CSDH: Chronic Subdural Hematoma
DOAC: Direct Oral Anticoagulants
GA: General anesthesia
GCS: Glasgow Coma Scale
LA: Local anesthesia
MGS: Markwalder Grading Scale
VKA: Vitamin- K antagonist.
